# A mixed methods needs assessment and gap analysis for establishment of a cancer research training program in East Africa

**DOI:** 10.29392/001c.22120

**Published:** 2021-04-29

**Authors:** Sarah Kutika Nyagabona, Beatrice Paul Mushi, Musiba Selekwa, Godfrey Sama Philipo, Sumaiya Haddadi, Emilie Fatima Kadhim, Lindsay Breithaupt, Sarah Maongezi, Julius Mwaiselage, Emmanuel Balandya, Germana Henry Leyna, Katherine Van Loon, Elia John Mmbaga

**Affiliations:** 1 Department of Epidemiology and Biostatistics, Muhimbili University of Health and Allied Sciences, Dar es Salaam, Tanzania; 2 Global Cancer Program, UCSF Helen Diller Family Comprehensive Cancer Center, San Francisco, California, USA; 3 Non-Communicable Diseases Program, United Republic of Tanzania Ministry of Health, Community Development, Gender, Elderly and Children, Dodoma, Tanzania; 4 Administrative services, Ocean Road Cancer Institute, Dar es Salaam, Tanzania; 5 Department of Epidemiology and Biostatistics, Muhimbili University of Health and Allied Sciences, Dar es Salaam, Tanzania; Department of Community Medicine and Global Health, University of Oslo, Oslo, Norway

**Keywords:** needs assessment, gap analysis, oncology, east africa, tanzania

## Abstract

**Background:**

The burden of non-communicable diseases (NCDs), including cancer, in Africa is rising. Policymakers are charged with formulating evidence-based cancer control plans; however, there is a paucity of data on cancers generated from within Africa. As part of efforts to enhance cancer research training in East Africa, we performed a needs assessment and gap analysis of cancer-related research training resources in Tanzania.

**Methods:**

A mixed-methods study to evaluate existing individual, institutional, and national resources supporting cancer research training in Tanzania was conducted. Qualitative data were collected using in-depth interviews while quantitative data were collected using self-administered questionnaires and online surveys. The study also included a desk-review of policy and guidelines related to NCD research and training. Study participants were selected to represent five groups: (i) policymakers; (ii) established researchers; (iii) research support personnel; (iv) faculty members in degree training programs; and (v) post-graduate trainees.

**Results:**

Our results identified challenges in four thematic areas. First, there is a need for coordination and monitoring of the cancer research agenda at the national level. Second, both faculty and trainees identified the need for incorporation of rigorous training to improve research competencies. Third, sustained mentoring and institutional investment in development of mentorship resources is critical to empowering early career investigators. Finally, academic institutions can enhance research outputs by providing adequate research infrastructure, prioritizing protected time for research, and recognizing research accomplishments by trainees and faculty.

**Conclusions:**

As we look towards establishment of cancer research training programs in East Africa, investment in the development of rigorous research training, mentorship resources, and research infrastructure will be critical to empowering local health professionals to engage in cancer research activities.

Non-communicable diseases (NCDs) pose increasing challenges to health systems throughout sub-Saharan Africa. It is projected that by 2030, 1.27 million new cancer cases and 0.97 million cancer-related deaths per year will occur in Africa.^1^ Most governments have declared NCDs, including cancer, to be among the major public health concerns that threaten economic development.^2,3^ In order to confront this mounting crisis, the World Health Organization (WHO) recommends the development of national cancer control plans that are systematic, equitable, and evidence-based.^4^ Although national policymakers are charged with formulating evidence-based cancer control plans, there is a paucity of data on the epidemiology, etiology, biology, and management of cancers from within Africa. Instead, many national cancer control plans are formulated based on data from high-income countries, which may not be pertinent in the African context.^5^

Over the past two decades, institutions in sub-Saharan Africa have made remarkable progress in building human research capacity. However, few African scientists who have achieved master’s or doctorate-level training are competitive for external funding sources.^6^ Data indicate that less than two percent of African research scientists have received two or more grants within 15 years after training.^7^ This is exemplified by disproportionately low research outputs and under-representation of African researchers as first and senior authors in research conducted in Africa.^6,8,9^

Most of the research output in Africa has focused on infectious diseases, with little attention to NCDs.^10^ Cancer research in East Africa is largely nascent. While higher learning institutions in Africa offer research training, there are notable deficiencies in research competencies among graduate clinicians in areas such as clinical research methods, cancer epidemiology, biostatistics, bioinformatics, and database management.^11^ As part of our ongoing effort to develop a sustainable cancer research training program in Tanzania, we performed a needs assessment and gap analysis of cancer-related research training resources.

## METHODS

### STUDY SETTING

Tanzania is a lower middle income country in East Africa with a population of 78 million people.^12^ The country’s health agenda is determined by the Ministry of Health Community Development Gender Elderly and Children (Mo-HCDGEC), which is responsible for establishing policies to guide preventive, clinical, and medical research practice for both communicable diseases and NCDs. The National Institute for Medical Research (NIMR) is responsible for setting and evaluating the national research agenda, and to monitor, control, and coordinate all medical research carried out within Tanzania. Muhimbili University of Health and Allied Sciences (MUHAS) is one of the oldest medical training and research institutions in East Africa and has trained over 75% of existing researchers and healthcare providers in Tanzania; it is currently the only university in Tanzania which provides formal training in clinical oncology. It also provides training for students from throughout eastern and central Africa. Ocean Road Cancer Institute (ORCI) is the nation’s specialized cancer hospital, providing the vast majority of chemotherapy and radiotherapy care to cancer patients in Tanzania. Muhimbili National Hospital (MNH) is the national referral hospital which provides a majority of pathological, radiological, surgical oncology, and pediatric oncology services in the country. Both ORCI and MNH are teaching hospitals affiliated with MUHAS.

### STUDY DESIGN

We conducted a mixed-methods study between March and June 2019 to evaluate individual, institutional, and national resources for cancer research training in Tanzania. Qualitative data were collected using in-depth interviews (IDIs) while self-administered questionnaires and online surveys were used to collect quantitative data. The study also included a desk-review of policy and guidelines related to NCD research and training at both national and institutional levels.

### STUDY POPULATION AND SAMPLING

Study participants were purposefully selected to represent five groups uniquely positioned to respond to needs assessment regarding cancer research.

1. *Policymakers* were asked to participate in IDIs. These policymakers included representatives from the NCD section and the Department of Policy and Planning at the MoHCDGEC and representatives from research units or directorates at NIMR, MUHAS, ORCI, and MNH responsible for the development and evaluation of institutional research agendas.
2. *Established researchers* from MUHAS, ORCI, and MNH with expertise in cancer research were selected to participate in IDIs to assess research capacity, needs, and prioritization.
3. *Research support personnel*, responsible for library services, information and communication, procurement, grant management, ethical reviews, and financial management at NIMR, MUHAS, ORCI, and MNH, were asked to participate in IDIs to assess existing research support and infrastructure.
4. *Faculty members in training degree programs* with components of training focused on research methodologies and/or cancer-related specialties were asked to complete self-administered questionnaires.
5. *Post-graduate trainees* enrolled in master’s programs in epidemiology, public health, or a clinical master’s program in a cancer-related specialty (e.g., pathology, oncology, radiology) were recruited to participate in an online survey.

### DATA COLLECTION AND ANALYSIS

IDIs, self-administered questionnaires, and online surveys were used to collect data for this study.

Survey tools were developed from previously validated tools including a previous survey tool used by researchers behind this manuscript. Following a systematic development process, the survey tools were pilot tested prior to data collection. Different qualitative data collection methods were utilized and involved different groups of participants with the aim of collecting wider views on the different themes and draw harmonized conclusions/opinions/suggestions.^11,13,14^

The tools aimed to collect data on four major thematic areas: (a) policies and research agendas; (b) research capacity and training needs; (c) mentorship resources; and (d) the research support environment.^11,15-20^

The IDI guides for policymakers were comprised of 18 open-ended questions focused on identifying national and institutional research needs, existing research strategies, and training. The IDI guides for established researchers were comprised of 23 open-ended questions that focused on the facilitators and barriers for career development in cancer research and included an inquiry into prior training, perceived competencies needed to conduct research, and the role of mentorship in research. The IDI guides for research support personnel were comprised of 30 open-ended questions that focused on the roles, current capacity, unmet demands for research and regulatory support, and future needs to support the development of these offices. Interviews for ethical review members focused on the frequency of the meetings, turnaround time, barriers faced, and suggestions for improvement.

The IDIs were conducted by five Tanzanian team members (SKN, EJM, BPM, ISM, GS), who had participated in the development of the interview guides. All the interviews were audio-recorded. IDIs were conducted in either English or Swahili, and conducted either in-person or via teleconference, depending on the preference of the participant.

Qualitative data were transcribed verbatim and translated into English, as needed then manually analysed by theme. Textual data were independently coded and analysed by three master’s degree graduates who were independent coders who had not participated in tools development or data collection to reduce risk of opinion bias. The coding system and analytic framework were based on both a priori concepts taken from the interview guides and key themes that emerged during manual open coding process. Findings and interpretations were iteratively compared among coders and reviewed by senior researcher (EJM) for harmonization. To validate the analysis, findings were presented and discussed with the whole study team and lastly shared by our collaborators from the ministry, ORCI and MUHAS prior to manuscript finalization.

The semi-structured questionnaire for faculty members had a total of 30 questions focused on self-evaluation of their professional development, competencies, personal research activities, and assessments of curricula and mentorship programs at MUHAS. The questionnaire aimed to understand experiences, knowledge, and attitudes towards cancer research and training, perceived barriers to conducting cancer research, and the adequacy of the existing training programs.

For the post-graduate trainees, the web-based questionnaire consisted of 46 open-ended questions that utilized different Likert scales to assess knowledge level. Questionnaire data were collected in REDCap™, with automatically generated email reminders sent to trainees at 7-day intervals to increase response rates. Descriptive analyses were performed using SPSS version 20. The results were organized according to four previously defined themes.

To evaluate existing national cancer control policies, research agendas, and research support, a review of NCD and cancer-related policies obtained from the MoHCDGEC and research agendas from NIMR, MUHAS, ORCI, and MNH was performed by three team members (BPM, EJM, and SKN).

### ETHICS CONSIDERATIONS

All individuals who participated in IDIs provided verbal consent to participate and for audio recording. All faculty participants gave verbal consent prior to being issued a questionnaire. Consent language was included in the invitational email for the web-based questionnaires, and consent was assumed by participants selecting the link to proceed to the questionnaire data that was collected and managed using REDCap™ electronic data capture tools hosted at [San Francisco Coordinating Center (SFCC)]. The study was considered exempt from review by the MUHAS Institutional Review Board (IRB).

## RESULTS

A total of 14 IDIs, 16 self-administered questionnaires, and 60 web-based questionnaires were completed with response rates of 88%, 53%, and 48%, respectively. The characteristics of the participants are summarized in Table 1. For the IDIs, the average length of an interview was 44 minutes (range 29 to 58 minutes). A total of four national and three institutional policies were reviewed.

**Figure 1.**
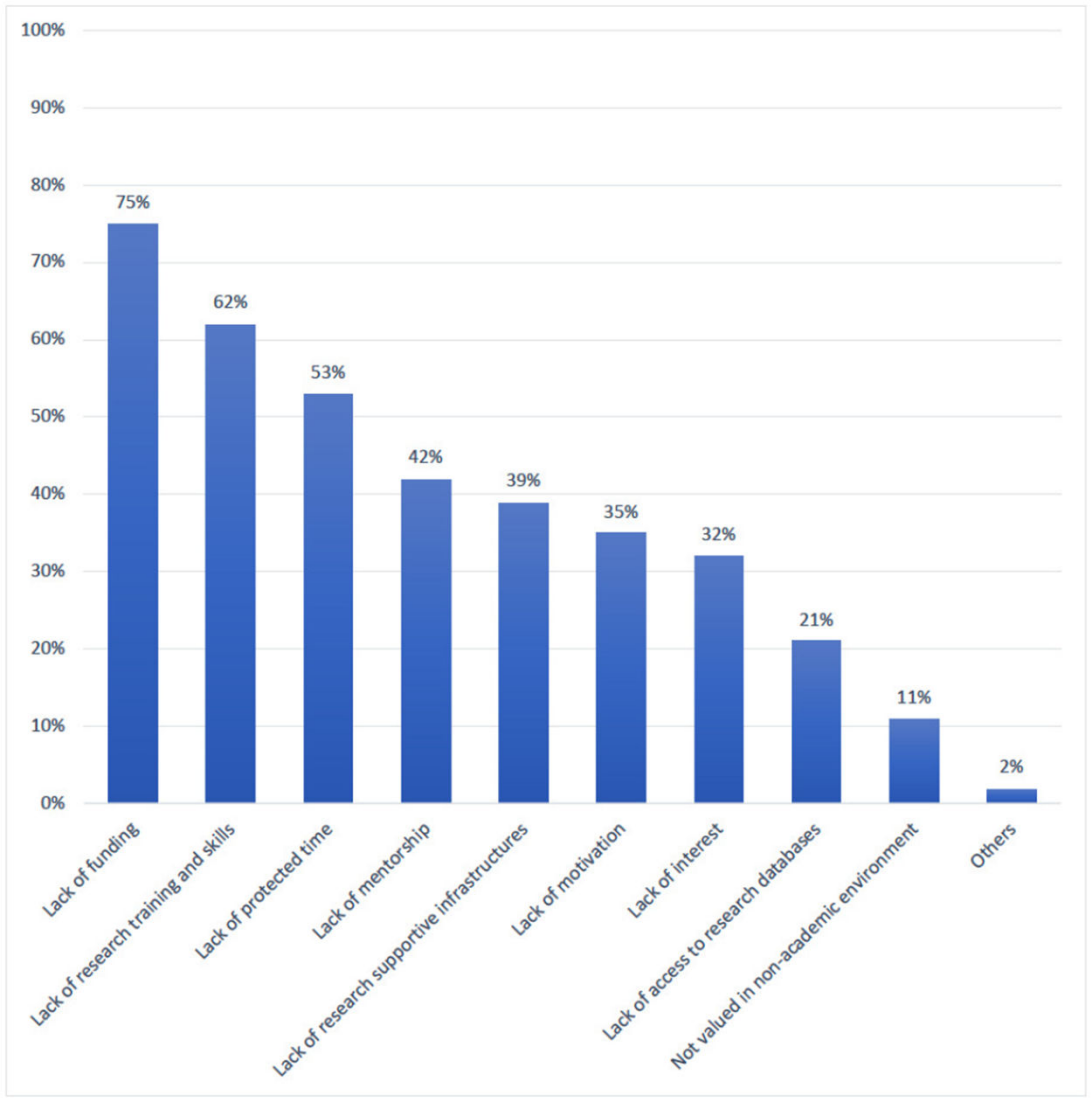
Barriers to involvement in research (in non-academic, clinical environment) by trainees at Muhimbili University of Health and Allied Sciences (N = 60).

Perceived reasons for the presence of limited research on NCD and cancer in Tanzania, included: (i) a scarcity of researchers in NCD and cancer; (ii) limited interest in research due to competing needs for financial resources; (iii) lack of research competencies; (iv) limited time for research due to teaching, administrative, and clinical care responsibilities; (v) absence of guidelines for protected time for research; and (vi) inadequate remuneration for research. Among post-graduate trainees, the top three barriers to research were: (i) inadequate funding (75%); (ii) inadequate research training and skills (62%); and (iii) inadequate protected time for research (53%) (Figure 1). Challenges reported by all participants are organized according to four thematic areas.

### THEME 1: THE NEED FOR COORDINATION OF NATIONAL CANCER POLICIES AND RESEARCH AGENDAS

The relevant national plans that support NCD and cancer research in Tanzania include: Research Priorities for Tanzania (COSTECH) (2015-2020),^21^ Health Sector Strategic Plan (HSSP IV) 2015-2020,^22^ Strategic and Action plan for Prevention and Control of Non Communicable Diseases in Tanzania (2016-2020),^23^ National Cancer Control Strategy (2013-2022),^24^ and institutional research agendas. These documents present research policies with priorities for the country and national institutions. We noted NCDs, such as cardiovascular diseases, diabetes mellitus, chronic respiratory conditions, and cancer were consistently among the top ten priority research areas in the national research agenda.

Policymakers, in particular, highlighted key priorities for NCD research in Tanzania (Table 2). They pointed out the need to strengthen coordination between research institutions in Tanzania, establish cancer surveillance programs and registries, and develop a national NCD-specific research agenda. While the national plans consistently promoted NCD research, the lack of an associated national budget to support research was mentioned as a significant barrier.

Respondents also highlighted the need to develop research monitoring framework and put in place mechanisms that promote the use of local research results in informing policies. The proposed research monitoring framework should aim at identification and coordination of research work among cancer researchers in the country to avoid duplication and promote collaboration among institutions and individual researchers.

Established researchers proposed a staff appraisal and promotion system that incorporates not only publications but also grant funding. They also highlighted the need for institutions to build opportunities for publication, dissemination of locally conducted research. In effort to address differences in capabilities and resources of institutions within Tanzania, they recommended development of guidelines for institutional sharing of resources.

### THEME 2: RESEARCH TRAINING

Established cancer researchers proposed priority cancer research topics across the continuum of cancer care in Tanzania and highlighted key competencies needed in the country to be able to conduct research. The specific competencies mentioned included research conceptualization, proposal development, and grant management.

With regards to competencies in teaching, only 25% (n=4) of faculty respondents ranked themselves as experts in teaching research, though a majority 93.8% (n=15) had participated in research development courses as learners. Self-identified strengths among faculty members included understanding of subject matter (93%), teaching large classes (62.5%), and development of assessment methods (62.5%). Meanwhile, use of audiovisual aids in teaching, information and computer technology skills, proposal development, data analysis and manuscript writing were identified as competencies that require more training support.

**Figure 2.**
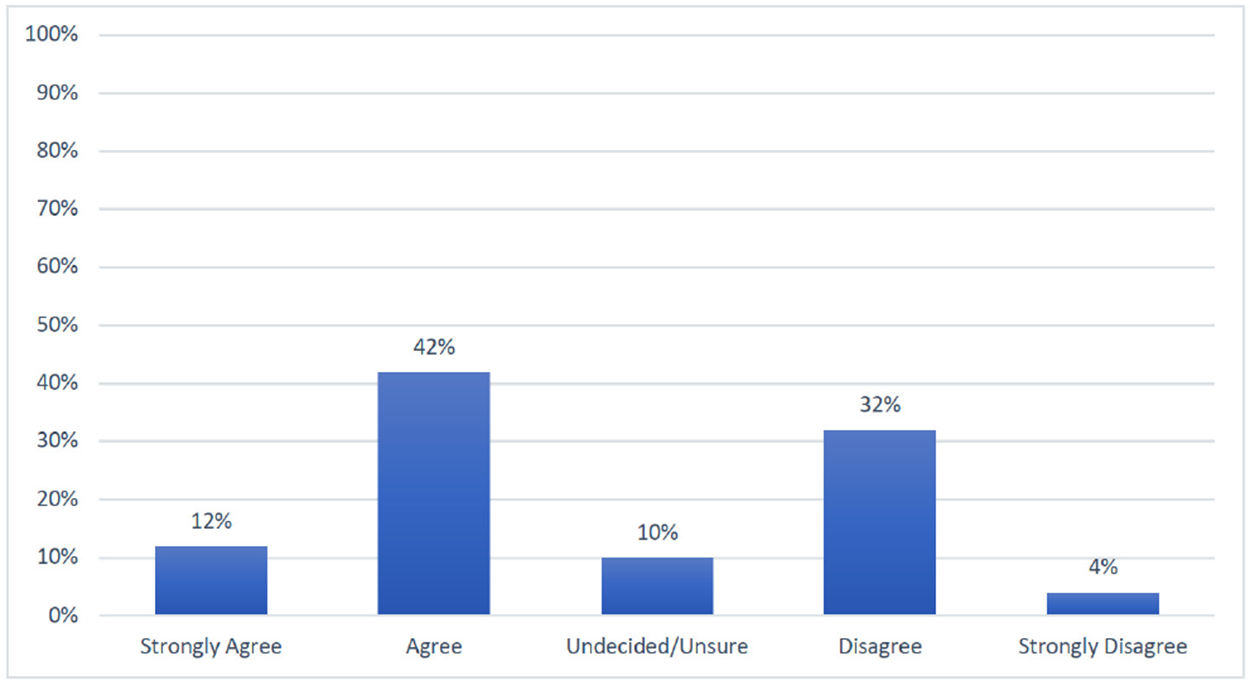
The research component of the existing curriculum in my current course is satisfactory in increasing competencies in cancer research? (N=60).

A majority (95%) of post-graduate trainees reported interest in incorporating research into their future careers. Despite the enthusiasm, (73%) also indicated that the existing master’s degree curriculums do not provide adequate training in research. In self-assessment of their own research knowledge and skills, most of the trainees self-reported only basic knowledge in research. About half of the respondents indicated that the current curriculum in their training course needs revision to be satisfactory in increasing competency required for conducting research (Figure 2). Specific topics recommended for inclusion included: (i) grant writing; (ii) biostatistical methods; (iii) clinical research methods; (iv) qualitative methods; (v) scientific writing and manuscript preparation; and (vi) presentation skills.

When asked regarding preferred training modalities, a majority of faculty expressed preferences for short term fellowships (37.5%) and short-term trainings (50%) which provide practical skills and also on-the-job mentorship (62.5%). Long-term didactic training was only desired by a smaller proportion of participants (31.3%). Established cancer researchers further suggested the need for sustained training approaches that are tailored for clinicians and adapted to competency improvement.

### THEME 3: MENTORSHIP

Established researchers consistently cited that strong mentorship has played an important role in achieving success as researchers. A majority of faculty members (75%) reported having mentors; however, they also reported that they had not received any training on mentorship principles (62.5%). All (100%) expressed the need for such training. Receipt of mentorship from experienced researchers was also consistently identified as important in training junior researchers. Of the trainees surveyed, 56% agreed with the statement that “Mentorship is important in research career development.” Despite these needs, half of the trainees (58%) either were undecided or disagreed with the statement “Getting mentors in your research topic of interest is easy.” Guidelines for mentorship exist at MUHAS, but not at MNH or ORCI. Of note, mentor-mentee pairings are typically made by administrative assignment and rarely form organically. Moreover, the intensity of the mentoring relationship relies heavily on the initiative of the mentee, and relationships are commonly perceived as unbalanced and uni-directional in terms of benefits. Finally, the interviews emphasized the overwhelming shortages in faculty with available bandwidth to provide mentorship. These shortages were attributed to lack of protected time for mentorship and competing clinical and academic responsibilities.

Participants provided suggestions of what is needed for an effective mentorship program. It was proposed for the institution to develop and disseminate mentorship guidelines that exist within a framework with measurable outcomes. It was also suggested that mentorship pairings could potentially improve if they are mutually driven, self-selected by the trainee-mentee pair, and grounded in shared interests between mentor and mentee. To improve capacity for mentoring, based on the results of this study, assigning protected time, and provision of incentives either financial compensation or institutional recognition (eg certificates or awards) were envisaged to have potential to build enthusiasm and morale.

### THEME 4: RESEARCH SUPPORT AND ADMINISTRATION

All institutions included in this assessment provide some administrative support for research. The supportive services offered by these offices range from before making a grant application to after one wins a grant award. The major challenges faced by the research support offices across all institutions were lack of adequate personnel within the units and limited information and communications technology resources to support researchers (computers, research management software, etc.).

The majority of faculty and researchers expressed needs for more support in procurement, financial management, and grant management. Research support personnel interviewed reported that there is a need for institutions to develop research support and resource guidelines that can be shared by all researchers. They also recommended inclusion of teaching on budgeting and financial management for researchers in the existing master programmes curriculum. This recommendation is based on the experience of the research support office supporting researchers in developing credible research budgets and financial management plans during project implementation.

The three institutions (MNH, MUHAS, and ORCI) have IRBs with mechanisms in place to grant permission for studies to be conducted in the institution’s premises. All research in the country requires an institutional ethical review and clearance. For those research grants which involve foreign investigators approval by the National Health Research Ethics Committee (NatHREC) is required. The main purpose of NatHREC approval is to protect and manage risk to human participants involved in research, ensuring ethical practice, prevent local data and specimen exploitation, and keep record of shared materials by different collaborating bodies.^25,26^

The MUHAS IRB supports ORCI and MNH and other domestic institutions with interest in medical-related research in Tanzania. The committee undertakes ethical and scientific review of all proposals. The average submission volume per month was about 50 research protocols, with an expected turnaround time of three weeks for expedited review and three months for a normal review. Quality of the submitted protocol was identified as another determinant of turnaround time. Furthermore, expedited vs. normal timeframes for review are associated with different costs to the investigator(s), and fees vary depending on whether the applicant is a student, local investigator, or an international collaborator.

Despite recent efforts to improve IRB functionalities to support research, several areas for improvement were identified, including: (i) a need for increased human resources to appropriately handle submission volumes; (ii) training researchers on proposal writing to improve the quality of submissions; (iii) disseminating guidelines for IRB proposal writing; (iv) developing a framework for post-award monitoring thus able to track study accruals, ethical compliance, renewals, and expirations; (v) electronic tracking of proposals; and (vi) updating IRB guidelines and standard operating procedures.

Established researchers also emphasized on the need for inter-institutional collaborations, so as to promote sharing of existing novel technologies that can be utilized in cancer research looking at etiological screening, diagnostics and treatment.

## DISCUSSION

Our study presents results from key cancer research stakeholders and describes the policy environment, researchers’ competencies, training gaps, and research support environment in Tanzania. The achieved response rates for in-person IDI was 88%, self-administered questionnaires, 53%, and for web-based questionnaires 48%. Response rates in research are better when person-person data collection methods are employed and relatively lower in self-administered or web-based approaches. This study achieved an excellent response rates for in-person interviews and although lower, our response rates from self-administered and web-based approach included about half of the participants, relatively better than other comparable studies. Weekly follow up reminders were issued in person for faculty and via email to trainees. These reminders were repeated three times before the participants were considered non-response. We however acknowledge the potential limitation and highlights challenge researchers face in response and attrition rate in research conduct.

Overall, the enthusiasm for cancer research is high; however, stakeholders unequivocally reported needs for training to build local capacity to effectively inform the revision and implementation of Tanzania’s national cancer control plan. Interviewed cancer researchers highlighted priority research areas of relevance along the cancer control continuum. However, limited research infrastructure, inadequate funding, lack of conducive environment for research, and a lack of national research coordination were identified as ongoing challenges. With each identified challenge, there is an opportunity for improvement and thus potential for improving future research development (Table 3)

NCD policies and strategies currently exist at the national level and at the national specialized hospitals and academic institutions in Tanzania. All reviewed policies primary focus on the need for human resources required to support prevention and clinical care activities for NCDs. While the National Cancer Control Strategy incorporates strategic objectives such as scaling up cancer surveillance, registries, cancer management, and prevention,^27^ the policy does not prioritize cancer research and does not provide a budget to facilitate research implementation. Moreover, the lack of a specific coordinating units for NCD research and management at the national level was highlighted by policymakers within the MoHCDGEC. Established researchers reported a growing need for coordination and monitoring of cancer research both at national and institutional levels in an effort to harmonize research efforts and to advocate for cancer research funding. Similar findings have been reported in other African countries, showcasing that locally funded training and research are critical in addressing issues in Africa’s research outputs.^28,29^

Deficiencies in research training were consistently reported by both faculty and trainees. These self-reported deficiencies are similar to findings reported by other African universities^30,31^ and result in the inability to train graduates that can conduct high-quality research. Post-graduate trainees expressed high motivation to incorporate research in their careers, although existing curriculums in terminal clinical degree programs do not emphasize research training. These findings echo our previous findings from an evaluation of oncology trainees in Tanzania,^11^ demonstrating that this disconnect prevails across post-graduate training programs. Lack of training in research skills has been reported to hinder clinicians’ involvement in research in both developed and developing countries,^32-34^ highlighting the need for dedicated research training programs.^33^ Proposed strategies to improve research capacity building among early career investigators included opportunities for tailored skill-specific short-term trainings (50%) and mentorship arrangements (62%).

Evidence from research and training institutions in Africa indicates strong mentorship is associated with increased research productivity.^35,36^ Experienced researchers interviewed in this study emphasized the role that mentorship has played in their careers and the need for sustained mentorship.

Mentorship guidelines at MUHAS did not provide a clear framework or measurable outcomes of mentoring relationships. The distinct expectations of a “Lead Mentor,” “Research Mentor,” and “Career Mentor,” which may be considered the norm^37^ in some academic settings, are not defined in the Tanzanian context. Blended roles are often attached to the single “Advisor” which is assigned by administration. In addition, there is a need for institutions to invest in development of mentorship resources, revision of existing guidelines to be more comprehensive with framework and outcome measures, faculty training on mentorship principles, and promotion of peer-to-peer mentorship among trainees.^38,39^

Lack of research recognition, protected time, and low remuneration for research and activities were identified by both faculty and established cancer researchers as barriers for research. This is consistent with a report from 27 African countries, which showed that a shortage of staff in research and academic institutions makes it difficult for academics and clinicians to dedicate time for research.^40^ Data from the Consortium for Advanced Research Training in Africa (CARTA) have also indicated that while researchers in the global south spend much time in research support, their research involvement is unrecognized and ill-compensated.^36^ These findings stress the need for fostering institutional environments to prioritize and support research within Africa. Participants in our study proposed non-monetary incentives for research productivity including credits in promotion evaluations, or opportunities to travel for the dissemination of research results. This theme highlights the inherent motivation to participate in research and provides concrete suggestions for modifications to the institutional environment and culture to support research works.

The effective conduct of research requires an adequate research support environment including pre-post award support, financial management, and timely ethical review. Our assessment identified the existence of frameworks for research support in all institutions, each with different levels of capacity and performance. While pre and post-award services exist, they are understaffed and under-resourced to support all researchers across disciplines. Similar to what has been reported in other countries in Africa, the lack of human resources, guidelines, poor internet connectivity, software, and computers compromise the effective performance of these research support mechanisms.^40^

All participating IRBs faced various challenges, including a lack of effective operating guidelines, limited human resources, and continued reliance on hardcopy submissions. In Tanzania, the efficiency of the IRB is slowed by persistent dependence on hardcopies and the national charge to conduct both ethical and scientific review of submitted applications. Improving the quality of work submitted will also allow for IRBs in Tanzania to improve efficiency by reducing time required for rigorous scientific review.

There is a need to mobilize local funds to support cancer research in Tanzania. Established cancer researchers emphasized that the focus of research funded by external sources is often determined by the funder or international collaborator, rather than by local priorities.^41^ Similarly, a review of research capacity in Africa has also shown that most available research training opportunities, including those supported by international institutions or donors, emphasize knowledge rather than research skills and do not prioritize sustainability.^42^ As we look to establish cancer research training pathways in Tanzania, we emphasize the need for local investment to ensure sustainability. While supports from international collaborations may serve to boost research in Africa, these collaborations should be formed as transparent and equitable partnerships, where both parties develop rules of engagement with clearly defined bidirectional benefits. Research priorities should be guided by local needs, and collaborative research should begin from the conception of research design with all involved parties with mutual respect and sharing experiences and knowledge.^43^

Pathology and other diagnostic and treatment technologies as well as new technologies and approaches for conducting novel clinical research vary by institutions in the country. However, the proposed inter-institutional collaborations for example the national laboratory, MNH and MUHAS which have some state-of-the-art research, diagnostics and treatment technologies has potential to improve research and diagnosis and treatment for better clinical outcomes. In order to capitalize on these collaborations there is a need for research training as the one proposed in this work, to be able to build a cadre of researchers who will be able to design and evaluate new technologies which are context specific and could be scaled up at low cost. We therefore hope the established research training will be able to foster and train a pool of mentors and clinical researchers in cancer and hence improve not only on the quantity but also the quality of cancer research with ultimate goal of impacting the cancer care continuum in the region and beyond.

## CONCLUSIONS

Cancer is contributing to an increased burden of disease throughout East Africa and has been named a priority area in Tanzania’s national research agenda. In November 2019, following completion of this analysis, the MoHCDGEC launched the NCD Program to oversee the planning, budgeting, implementation, and coordination of NCD.^44^ This marks an important step towards the establishment of a national coordination unit for NCD and cancer research in Tanzania and reflects that cancer research is indeed a national priority in Tanzania. As we look towards the establishment of a D43-funded cancer research training program in Tanzania, investment in the development of rigorous research training, mentorship resources, and research infrastructure will be critical components towards empowering local health professionals to pursue high quality research activities. While the motivation to pursue careers in cancer research is high amongst both academic faculty and trainees in Tanzania, there is a critical need for national and institutional leaders to explore strategies and define metrics for invoking changes in clinical and academic culture to ensure and enforce protected time for research and to emphasize and reward research productivity.

## Figures and Tables

**Table 1.** Characteristics of participants

Position	Participants	Institution/ affiliation	Department(s)	Methodology
Policymakers	1	MoHCDGEC	NCD Program Policy and Plan	In-depth interviews
	1			
Established researchers	1	MNH, ORCI, MUHAS	Internal Medicine Clinical Oncology	
	4			
Research support personnel	4	MUHAS, MNH, NIMR	Research and Publication Financial management services Library services Information and communications technology Institutional Review Boards	
	2			
Faculty members in training degree programs	7	MUHAS, ORCI	Behavioral sciences Epidemiology and Biostatistics Pathology Radiology Clinical oncology	Self-administered questionnaire
	9			
Post-graduate trainees	27	MUHAS	**Master of Science (MSc) in:** Applied Epidemiology Applied Laboratory in Epidemiology **Master of Medicine (MMed) in:** Pathology Radiology Clinical oncology	Electronically administered questionnaire
	33			

NCD–Non-communicable diseases, NIMR–National Institute for Medical Research, MNH–Muhimbili National Hospital, MoHCDGEC–Ministry of Health Community Development Gender Elderly and Children, MUHAS–Muhimbili University of Health and Allied Sciences, ORCI–Ocean Road Cancer Institute.

**Table 2.** Quotes by theme

Theme	Quote	Summary
Theme 1	“…..You know NCD magnitude is increasing, [it is] becoming a big problem and the Ministry has plans to establish an NCD program from November 2019. This will increase visibility and probably ensure that we have a clear and separate budget to implement our plans…”	Establishing a formal plan will support visibility and clarity.
Theme 1	“…in research, it’s difficult to predict which area will be implemented and also monitored; because [this] depends on funding …. Sometimes nothing is done in some areas sometimes so much is done but in a non-coordinated manner……”	Funding determines research areas.
Theme 1	“… we do not know who is doing what and where, you go into an international conference and you hear people present results of cancer studies in Tanzania, you are [surprised] like ooh….	There is a lack of communication regarding ongoing research.
Theme 1	“In collaboration … you cannot work in insolation. So, if ORCI works together with MUHAS, NIMR, TMDA, MNH, and the national referral laboratory, I'm sure we can implement many studies… even a clinical trial.”	Given that research institutions in Tanzania have different capabilities and resources and there is a need for sharing of resource across institutions.
Theme 1	“My recommendation is: maybe we need to look again on the current practice and reorganize in such a way that we can efficiently utilize time better, and also maybe if our promotion can depend on research like the number of publications or number of grants and hence this can be translated or applied and is included as part of your work performance.”	There is a critical need for national and institutional leaders to explore strategies and define metrics for invoking changes in clinical and academic culture to ensure and enforce protected time for research and to emphasize and reward research productivity.
Theme 2	“…. the workshops that we usually conduct for a week or two don’t seem to be working. The [international] researchers train and go, and usually nothing materializes …. We need to have a follow-through and continuous support for junior staff and those who are not really well experienced in research.”	Sustainability should be emphasized in development of training efforts.
Theme 3	“…. the institution has a shortage of staff; I think more than 40% deficit in skilled staff. So… protected time for mentoring and conducting research is not practical in the current situation.”	Staff shortages contribute to lack of available mentors.
Theme 3	“You cannot be a good researcher without mentorship. Mentors help to build your skills by providing the opportunity to do the actual work and also linkage to collaborators and donors….”	The importance of mentorship is highlighted.
Theme 3	“We don’t have critical mass of mentors, so we have few identified people who are willing and have done work in research. But they are not many in number, so it is difficult to say we have a robust mentorship program…”	There are few independent researchers who are capable of providing mentorship.
Theme 3	“Everybody needs to be mentored at some point. Nobody can work well without mentorship…	The importance of mentorship is highlighted.
Theme 3	“I tell them to see me twice a week, and they should not expect me to follow them. They should come…	The mentoring relationship relies on the initiative of the mentee.
Theme 3	“…. in some courses the coordinator may appoint just anyone to mentor students, in others it will be the task of a student to look for a mentor that they feel is appropriate for them, and I support that kind of a model more than being a prescription…I usually don’t prefer students being prescribed to me, I have never had good experiences with those kinds of students. But those students who come to me [organic pairing], we set time of reference from the very first day…”	In the mentor and mentee relationship it is important to establish a bi-directional relationship.
Theme 3	“…. by making it mandatory for them to engage in research and publications, [research] can actually guarantee their promotion at work. Thus, there is no promotion without publications, we need to actually give equal weight for teaching as well as research.”	Institutional culture and promotion policies may increase motivation for research.
Theme 4	“.we don’t have a centralized space which has all available resources people can use. So, in terms of infrastructure it is not yet well established for research.”	There is a lack of adequate resources and infrastructure for research.
Theme 4	“The researchers sending these applications are scientists, experts in that area but when it comes to legal aspects, financial aspects, checking the budget to see if it will sustain the next five years, making sure that everything that is required during the implementation of the project… it is very easy to overlook something.”	Researchers need training in project management.
Theme 4	“In terms of the infrastructure, I can say; looking at ORCI it lacks some of the highly specialized equipment but the National laboratory has a lot of facilities and this is equipped for conducting research. The main issue is using/sharing of resources”	Need for inter-institution collaboration to foster quality of research done

NCD–Non-communicable diseases, NIMR–National Institute for Medical Research, MNH–Muhimbili National Hospital, MUHAS–Muhimbili University of Health and Allied Sciences, ORCI–Ocean Road Cancer Institute, TMDA–Tanzania Medicines and Medical Devices Authority.

**Table 3.** Themes and opportunities for a cancer research training in Tanzania

#	Themes	Opportunity
1	The need for coordination of national cancer policies and research agendas	Inter-institutional collaboration
2	Research training	Development of cancer research training program at MUHAS
3	Mentorship	Mentorship frame Identify and train pool of mentors within the MNH, ORCI and MUHAS institutions
4	Research support and administration	National and Institutional funding for research

MNH–Muhimbili National Hospital, MUHAS–Muhimbili University of Health and Allied Sciences, ORCI–Ocean Road Cancer Institute.
